# Protein S Deficiency and the Risk of Venous Thromboembolism in the Han Chinese Population

**DOI:** 10.3389/fcvm.2021.796755

**Published:** 2022-06-23

**Authors:** Yingying Wu, Jingdi Liu, Wei Zeng, Bei Hu, Yu Hu, Liang V. Tang

**Affiliations:** ^1^Department of Oncology, Tongji Hospital, Tongji Medical College, Huazhong University of Science and Technology, Wuhan, China; ^2^Institute of Hematology, Union Hospital, Tongji Medical College, Huazhong University of Science and Technology, Wuhan, China; ^3^Hubei Clinical and Research Centre of Thrombosis and Haemostasis, Wuhan, China; ^4^Department of Radiation and Medical Oncology, Zhongnan Hospital, Wuhan University, Wuhan, China

**Keywords:** protein S deficiency, venous thromboembolism, gene mutation, odds ratio, Chinese population

## Abstract

Plasma levels of the anticoagulant cofactor protein S and PROS1 mutation are reported to impart increased risk of thromboembolism in European and south east Asian populations, but the relationship is not yet documented in Han Chinese in population-based study. Therefore, we undertook a case-control study of this relationship among patients with venous thromboembolism, and probed the genetic factors contributing to low protein S deficiency. Among the 603 consecutively recruited venous thromboembolism patients, 51 (8.5%) proved to be deficient in free protein S antigen (lower than 38.6 U/dl), among whom 30 cases were identified to have a causative mutation by direct sequencing. In contrast, six cases (1.0%) of the 584 healthy controls had low free antigen levels, among whom direct sequencing confirmed disease-causing gene mutations in four controls (0.7%). After adjusting for age and gender, the odds ratio of developing venous thromboembolism in individuals with protein S deficiency based on free protein S tests was 8.1 (95% CI = 3.6–19.9, *P* < 0.001). Gene sequencing yielded 24 different heterozygous mutations in the 34 participants, of which 13 were newly described. 17 (50%) of the 34 mutations in our study cohort occurred in exons 12 and 13, indicating the LGR2 domain to be a hotspot mutation region for the protein. These findings are conducive to the clinical application of protein S assays for the molecular diagnosis of thrombophilia.

## Introduction

Protein S is a natural vitamin K-dependent plasma glycoprotein (1, 2). Protein S acts as a cofactor of anticoagulant protease, activated protein C (APC), which inactivates the procoagulant factor Va and VIIIa to inhibit thrombin generation for regulating of coagulation (3). Protein S also has an APC-independent anticoagulant effect in inhibiting prothrombinase and the factor Xase (FXa) complex (4). Recent evidence suggests that protein S has yet another anticoagulant activity through interaction with the tissue factor pathway inhibitor (TFPI) to inhibit FXa (5, 6). In plasma, ~60% of circulating protein S is bound to C4b-bining protein (C4BP), while the remaining 40% free fractions possesses APC cofactor activity (7).

Inherited protein S deficiency (MIM 176880) is a rare autosomal dominant disorder due to various mutations in the PROS1 gene, which maps to human chromosome 3q11.2, spanning 101 kb and comprising 15 exons (8). The prevalence of protein S deficiency in healthy population reportedly broadly ranges from 0.03 to 0.13% in healthy European healthy populations, but increases to 1–13% in patients diagnosed with venous thromboembolism (9–11). New studies uncover that protein S deficiency has a relatively higher prevalence in southeast Asian general populations and patients with venous thromboembolism (12–14). Family-based studies reveal that protein S deficiency predisposes individuals to suffer venous thromboembolism, whereas the population-based studies indicate conflicting results on this association (15, 16). In addition to congenital mutations (17), oral contraceptives, pregnancy, hormone-replacement therapy, hypoxia and hepatic disorders can also decrease protein S levels (18–20).

Despite this background, there has been no study hitherto investigating the association between protein S deficiency and venous thromboembolism risk in the Chinese population. Therefore, we undertook a case-control study of the relationship between low plasma levels of free protein S, PROS1 mutation and increased risk of venous thromboembolism in a Chinese population. At the same time, we described the genetic characters of protein S deficiency in our study population.

## Methods

### Subjects

A total of 603 unselected patients diagnosed with symptomatic venous thromboembolism were consecutively enrolled in the study from 1 January 2014 to 12 December 2015 in Wuhan Union Hospital. The criteria for diagnosis were according to clinical manifestations, D-dimer, and imaging tests. Color Doppler Ultrasonography, Computed Tomography angiography or Magnetic Resonance venography were performed in participants with symptomatic venous thromboembolism. Five hundred eighty-four age- and sex-matched controls without an individual history of venous thromboembolism were recruited during the same period. Blood samples were drawn, immediately centrifuged, the platelet-poor plasma samples were stored at −80°C for protein S antigen assays. Separated white blood cells were extracted for genomic DNA analysis.

This study was approved by ethics committee of Union hospital affiliated to Hua Zhong University of science and technology. All methods performed in our study were exactly in accordance with the approved guidelines. Written informed consent in compliance with the Declaration of Helsinki was obtained from all participants or their legal guardians.

### Sample Collection and Plasma Free Protein S Assay

Sample were collected before anticoagulant therapy or at 2 weeks after its discontinuation of anticoagulant therapy. Free protein S antigen was measured by enzyme-linked immunosorbent assay using the ZYMUTEST Free Protein S kit, following the manufacturer's instructions (Hyphen BioMed, Andresy, France).

### The *PROS1* Gene Analysis

The putative promoter, 5'UTR, 15 exons and their flanking regions, and 3'UTR were amplified though the Polymerase Chain Reaction (PCR) and reaction products were sequenced on an ABI PRISM 3730XL automated sequencer (Applied Biosystems). The primer-pair sequences and PCR conditions used in the amplification were as described in a previous report (21). We described novel variants according to current nomenclature conventions and the recommendations of the Human Genome Variation Society (HGVS, http://www.hgvs.org/mutnomen/). The Genebank NM_000313.3 and NP_000304.2 were used as reference sequence. Functional consequences of novel missense mutations were analyzed by *in silico* bioinformatics tools. MutationTaster: http://www.mutationtaster.org/; PROVEAN: http://provean.jcvi.org/index.php; PolyPhen-2: http://genetics.bwh.harvard.edu/pph2/; HomoloGene for PROS1: https://www.ncbi.nlm.nih.gov/homologene/264

### Statistical Analysis

The odds ratios and 95% confidence intervals (95% CIs) evaluating the risk of venous thromboembolism associated with the plasma level of free protein S antigen or PROS1 mutations were calculate through the Chi-squared test. Multivariate logistic regression analysis was performed to adjust the odds ratios (OR) for selected confounders (age and gender). Statistical significance was accepted at *P* < 0.05. The statistical analyses were carried out using SPSS 19.0 (SPSS Inc., Chicago, IL, USA).

## Results

### Characteristics of Participants

**Figure 1:**
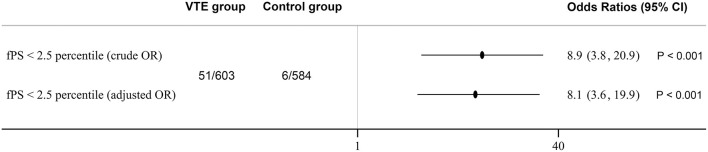
Protein S deficiency and risk of venous thromboembolism. Odds Ratios were adjusted for age and gender. 95% CI indicates 95% confidence intervals.

The population consisted of 603 consecutive venous thromboembolism patients and 584 controls. Female and male had similar mean free protein S level, as shown along with other results in Table 1. We established a local laboratory free protein S reference range from previous healthy individuals, whereby plasma concentrations falling below the 2.5th percentile (38.6 U/dl) were defined as protein S deficient. Among the 603 consecutively recruited venous thromboembolism patients, 51 (8.5%) proved to be deficient in free protein S antigen, among whom 30 cases were identified to have a causative mutation by direct sequencing. Six cases (1.0%) of the 584 normal controls had free antigen levels lower than 38.6 U/dl, among whom direct sequencing confirmed disease-causing gene mutations in four controls (0.7%). As shown in Figure 1, the OR of venous thromboembolism in individuals with protein S deficiency based on free protein S tests was 8.9 (95% CI = 3.8–20.9, *P* < 0.001), and 7.6 (95% CI = 2.6–21.7, *P* < 0.001) in individuals with protein S deficiency based on PROS1 gene mutations. After adjusting for age and gender, the odds ratio of developing venous thromboembolism in individuals with protein S deficiency based on free protein S tests was 8.1 (95% CI = 3.6–19.9, *P* < 0.001).

**Table 1:** Free protein S levels grouped by statistic characteristics.

**Characteristics**	**VTE patients**	**Healthy controls**
	**(means of fPS level: U/dl**	**(means of fPS level: U/dl**,
	***N =* 603)**	***N =* 584)**
**Age (years)**
<20	73 ± 17, *n =* 8	97, *n =* 1
20~40	84 ± 18, *n =* 89	87 ± 13, *n =* 113
40~60	89 ± 23, *n =* 295	89 ± 13, *n =* 304
>60	89 ± 20, *n =* 211	87 ± 13, *n =* 166
**Gender**
Male	87 ± 20, *n =* 324	87 ± 13, *n =* 292
Female	88 ± 23, *n =* 279	88 ± 13, *n =* 292
**Current smoking**
Yes	90 ± 16, *n =* 118	87 ± 13, *n =* 116
No	87 ± 23, *n =* 485	88 ± 13, *n =* 468
**Alcohol drinking**
Yes	87 ± 18, *n =* 75	84 ± 19, *n =* 64
No	88 ± 22, *n =* 528	84 ± 23, *n =* 520
**History of VTE**
Yes	84 ± 18, *n =* 72	NA
No	88 ± 22, *n =* 531	NA
**Malignant tumor**
Yes	91 ± 13, *n =* 14	92 ± 12, *n =* 3
No	88 ± 22, *n =* 589	88 ± 13, *n =* 581
**Pregnancy/puerperium**
Yes	77 ± 7, *n =* 5	80 ± 18, *n =* 13
No	88 ± 22, *n =* 598	88 ± 13, *n =* 571
**Recent surgery**
Yes	88 ± 17, *n =* 36	81 ± 17, *n =* 8
No	88 ± 22, *n =* 567	88 ± 13, *n =* 576

*Recent surgery: refers to receiving surgery within 3 months*.

### Genetic Analysis of *PROS1*

To study the genetic molecular basis of low free protein S levels, we performed DNA sequencing of the *PROS1* gene in participants with free protein S level below our cut-off of 38.6 U/dL; this yielded sequence information in 51 patients with venous thromboembolism and 6 controls. In 34 of 57 sequenced participants with PS deficiency, we found a total of 24 heterozygous gene mutations, including 13 new mutations (Table 2). Four heterozygous mutations were detected in the healthy control PS-deficiency subjects. The positive rate of genetic analysis might be due to the occurrence of a causative variant in deep introns that were overlooked by the DNA sequencing, or due to gross insertion/deletion of the *PROS1* gene.

**Table 2:** Molecular analysis of PROS1 in participants with free protein S below the 2.5 percentile.

**Participant ID**	**Age**	**Sex**	**fPS (U/dL)**	**NT exchange**	**AA substitution**	**Region**	**Domain**	**Newly reported**
VTE 1	57	M	38	c.1454A>C	p.Tyr485Ser	E12	LGR2	Yes
VTE 2	68	M	33	c.829C>T	p.Gln277*	E8	EGF4	Yes
VTE 3	42	F	23	c.1424G>A	p.Cys475Tyr	E12	LGR2	Yes
VTE 4	50	M	36	c.200A>C	p.Glu67Ala	E2	GLA	rs766423432
VTE 5	61	F	19	c.1393G>T	p.Glu465*	E12	LGR2	rs199469496
VTE 6	43	F	29	c.1543C>T	p.Arg515Cys	E13	LGR2	rs199469500
VTE 7	40	F	30	c.200A>C	p.Glu67Ala	E2	GLA	rs766423432
VTE 8	61	M	30	c.1063C>T	p.Arg355Cys	E10	LGR1	rs387906674
VTE 9	22	M	34	c.1351C>T	p.Arg451*	E12	LGR2	rs5017717
VTE 10	44	M	37	c.200A>C	p.Glu67Ala	E2	GLA	rs766423432
VTE 11	64	M	33	c.1543C>T	p.Arg515Cys	E13	LGR2	rs199469500
VTE 12	52	M	34	c.1603T>G	p.Phe535Val	E13	LGR2	Yes
VTE 13	54	F	35	c.1565T>A	p.Val522Asp	E13	LGR2	Yes
VTE 14	49	M	28	c.203C>A	p.Ala68Asp	E2	GLA	No
VTE 15	59	F	29	c.1146_1147delAT	p.Trp383Glufs*11	E10	LGR1	rs312262905
VTE 16	70	M	38	c.1351C>T	p.Arg451*	E12	LGR2	rs5017717
VTE 17	39	M	32	c.1543C>T	p.Arg515Cys	E13	LGR2	rs199469500
VTE 18	54	F	21	c.1518G>A	p.Trp506*	E13	LGR2	No
VTE 19	50	M	29	c.483C>A	p.Cys161*	E6	EGF2	Yes
VTE 20	34	F	30	c.1577T>G	p.Leu526Trp	E13	LGR2	Yes
VTE 21	40	M	34	c.1543C>T	p.Arg515Cys	E13	LGR2	rs199469500
VTE 22	35	M	27	c.1704T>A	p.Cys568*	E14	LGR2	Yes
VTE 23	18	F	17	c.365_366delGT	p.Ser122Thrfs*6	E5	EGF1	Yes
VTE 24	61	F	17	c.1229C>A	p.Pro410His	E11	LGR1	rs199469495
VTE 25	24	M	20	c.976G>T	p.Glu326*	E10	LGR1	Yes
VTE 26	50	M	33	c.1351C>T	p.Arg451*	E12	LGR2	rs5017717
VTE 27	62	F	37	c.1543C>T	p.Arg515Cys	E13	LGR2	rs199469500
VTE 28	46	M	34	c.1553C>T	p.Thr518Met	E13	LGR2	rs373336653
VTE 29	52	M	29	c.392A>G	p.Tyr131Cys	E5	EGF1	Yes
VTE 30	67	F	29	c.1095T>G	p.Asn365Lys	E10	LGR1	rs199469491
HC 1	64	F	31	c.1063C>T	p.Arg355Cys	E10	LGR1	rs387906674
HC 2	44	F	28	c.1155+4C>T	Splicing region	E10	LGR1	Yes
HC 3	22	F	20	c.282_282delT	p.Leu96Tyrfs*15	E4	TSR	Yes
HC 4	29	M	31	c.1543C>T	p.Arg515Cys	E13	LGR2	rs199469500

Mutations were designated according to the human Genome Variation Society compared with NCBI Reference Sequences NM_000313.3 and NP_000304.2. VTE, refers to venous thromboembolism patients; HC, refers to healthy control individuals; E, exon; fPS, free protein S antigen; NT, nucleotide; AA, amino acid; E, exon; I, intron; EGF, epidermal growth factor domain; TSR, thrombin-sensitive region; GLA, γ-carboxyglutamic acid domain; LGR, laminin G-type repeat domain; ^*^nonsense mutation; M, male; F, female.

We evaluated the 6 newly discovered *PROS1* gene missense mutations for pathogenicity through four functional prediction software procedures, at least three of which indicated pathogenicity for all mutations (Table 3). We did not evaluate further small deletions, non-sense mutations and previously reported detrimental variants. Four of the controls carried a variant that was identified as distinctly damaging, despite the absent phenotype of venous thromboembolism. This may be explained by that venous thromboembolism is doubtless a multifactorial disease caused by interactions between genetic and environmental factors; the controls carrying an abnormal genotype may be vulnerable to venous thromboembolism later in life, or upon exposure to some environmental factor.

**Table 3:** *In silico* analysis of novel amino acid changes.

**NT exchange**	**AA substitution**	**MutationTaster**	**PROVEAN**	**PolyPhen-2**	**HomoloGene**
c.392A>G	p.Tyr131Cys	Disease causing (probability: 0.996)	Neutral	Probably damaging (probability: 0.993)	Highly conserved
c.1424G>A	p.Cys475Tyr	Disease causing (probability: 0.999)	Deleterious	Probably damaging (probability: 0.993)	Highly conserved
c.1454A>C	p.Tyr485Ser	Disease causing (probability: 0.999)	Deleterious	Possibly damaging (probability: 0.914)	Highly conserved
c.1565T>A	p.Val522Asp	Disease causing (probability: 0.999)	Deleterious	Probably damaging (probability: 0.997)	Highly conserved
c.1577T>G	p.Leu526Trp	Disease causing (probability: 0.999)	Deleterious	Probably damaging (probability: 0.999)	Highly conserved
c.1603T>G	p.Phe535Val	Disease causing (probability: 0.601)	Neutral	Possibly damaging (probability: 0.925)	Highly conserved

*Functional consequences of novel missense mutations were analyzed by in silico bioinformatics tools. MutationTaster: http://www.mutationtaster.org/; PROVEAN: http://provean.jcvi.org/index.php; PolyPhen-2: http://genetics.bwh.harvard.edu/pph2/; HomoloGene for PROS1: https://www.ncbi.nlm.nih.gov/homologene/264; NT, nucleotide; AA, amino acid*.

**Figure 2:**
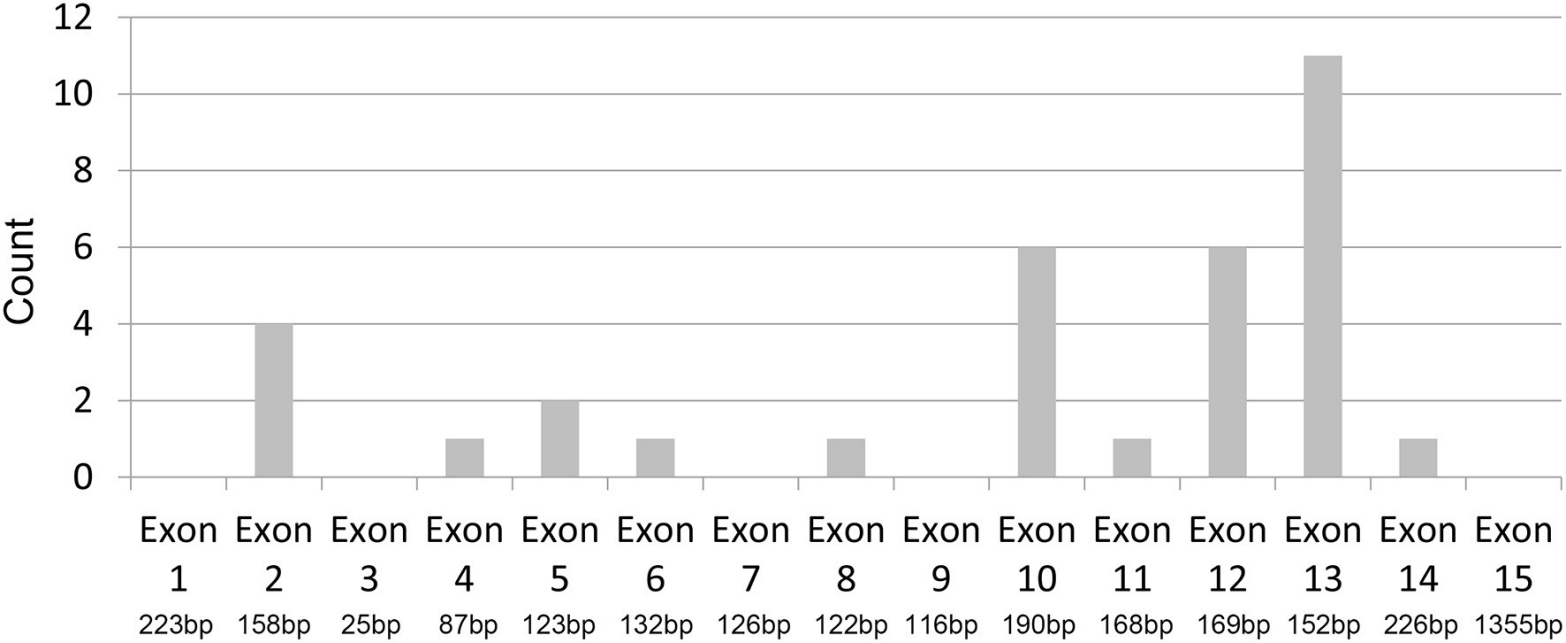
Hot-spot region of protein S mutation. Distribution of PROS1 mutations across exon 1 to 15: 50% (17/34) of the identified mutations occurred in exons 12 and 13.

We were surprised to find that among the 34 participants with identified gene mutations, 11 were in exon 13 and six were in the adjacent exon 12, comprising part of the LGR2 domain of the protein. Thus, 17 (50%) of the 34 mutations in our study cohort occurred in the exons 12/13, indicating a hotspot mutation region of protein S (Figure 2).

## Discussion

Risk factors and epidemiology of venous thromboembolism have been extensively studied in Caucasians populations during the past years. However, the corresponding state of knowledge concerning incidence and genetic factors lags behind in Asian populations. There is compelling evidence for racial and geographic differences in the hereditary risk factor and incidence of venous thromboembolism. Gain-of-function variations in procoagulant factors was the main genetic risk factor for venous thromboembolism in Caucasians, while loss of function of anticoagulation factors was the predominant cause in Asians. Specifically, deficiency of the natural anticoagulant factors protein S, protein C and antithrombin were common risk factors for venous thromboembolism in China. Among these, protein S deficiency was the more frequently compromised among the anticoagulant factors.

Protein S deficiency was established early to be risk factor associated with familial venous thromboembolism in European populations (22), as substantiated by extensive family-based studies (23). Thrombophilic subjects were 5–10-fold more likely to harbor a protein S deficiency compared to healthy relatives (24–26). Nevertheless, there have been discordant findings concerning the risk of venous thromboembolism on protein S deficiency in population-based studies. We now show that a low level of free protein S antigen imparted an increased risk of developing venous thromboembolism in a Han Chinese population, which stands in agreement with some previous research (27, 28). We wish now to emphasize some aspects of the conflicting results in the literature on protein S deficiency. First, the accurate, reproducible and reliable diagnosis of protein S deficiency is technically challenging (29). One prior study reported that only nine of 56 cases with initially low protein S antigen levels showed persistence of their decreased values, thus indicating that low findings are often a transient phenomenon (30). Furthermore, transient protein S deficiency does not seem sufficient in impart increased risk of venous thromboembolism. Second, protein S assay results differ by region, instrumentation, and clinical laboratory. Third, determining the association with risk of venous thromboembolism depends on measuring the level of protein S antigen accurately and reproducibly in reference to the normal range for the local population. Indeed, protein S antigen levels are affected not just by hereditary, but due to factors such as smoking, surgery, disease, pregnancy/puerperium, age, and sex (31). Fourth, there is some overlap of free protein S levels between healthy individuals and hereditary protein S deficiency patients with a history of venous thromboembolism (32, 33).

In the present study, diagnosis of protein S deficiency relied on the results from laboratory tests measuring protein antigen level. In our hands, the free protein S antigen assay was more accurate, reproducible, and valuable for identifying protein S deficiency status, than were the total protein S antigen assay and protein S activity test. The widely used activity assay measuring the anticoagulant function of protein S as the cofactor of APC is fraught with problems causing spuriously false low protein S values (34). Total protein S, consisting of the bound and free protein S fractions measured immunologically was not associated with risk of venous thromboembolism (35). Indeed, some individuals with hereditary and acquired protein S deficiency may manifest normal total protein S levels, while harboring decreased free protein S antigen and declining protein S activity levels (36). We performed the free protein S antigen assay to immunologically recognize only the unbound form of protein S. In previous work, the proportion of falsely low values of free protein S antigen (<1%) was distinctly lower than in activity assays (10–15%), indicating that free protein S antigen assay is a more accurate and reliable method evaluating the protein S functional state (37).

Heterozygous protein S deficiency is well-established as an autosomal dominant trait associated with an increased risk for developing venous thromboembolism (38, 39). Missense mutations, non-sense mutations and small insertion/deletions accounted the majority of defects in the present study. The latter two mutations were obviously detrimental because they contained a premature stop codon causing a truncated protein with impaired function. In this study, at least three of four *in silico* bioinformatic tools predicted the novel missense mutations to be harmful.

Disease-causing mutations were not identified in a large proportion of our protein S deficient subjects. This may arise due to presence of a mutation located in deep introns that are invisible to the DNA sequencing method, or due to the presence of large insertion/deletion mutations, which are a rare risk factor for developing venous thromboembolism (39). Our results were in accordance with other research showing an undetected mutation of the *PROS1* gene in up to 50% of individuals with protein S deficiency (8). On the other hand, diagnosis of protein S deficiency remains a challenge due to the variable of possible genetic defects, assay performance, and various confounds such as use of oral contraceptives, surgery, infection, DIC, pregnancy/puerperium, HIV infection, and liver disease.

In summary, we investigated the effects protein S deficiency (based on plasma levels for the free antigen) and *PROS1* gene mutations in a series of patients of Han ethnicity. We found a 6–8-fold elevated risk for venous thromboembolism among patients with protein S deficiency, and furthermore discovered a mutation hotspot around exons 11 and 12 of *PROS1*. These findings are conducive to the clinical application of protein S assays for the molecular diagnosis of thrombophilia.

## Data Availability Statement

The data presented in the study are deposited in the NCBI repository, accession number is ON730818, ON730819, ON730820, ON730821, ON730822, ON730823, ON730824, ON730825, and ON730826.

## Ethics Statement

The studies involving human participants were reviewed and approved by Ethics Committee of Union Hospital Affiliated to Hua Zhong University of Science and Technology. Written informed consent to participate in this study was provided by the participants' legal guardian/next of kin. Written informed consent was obtained from the individual(s), and minor(s)' legal guardian/next of kin, for the publication of any potentially identifiable images or data included in this article.

## Author Contributions

YW contributed to the study design. YW and JL collected samples, performed the experiment, and analyzed the data. LT and YH wrote and revised the manuscript. All authors contributed to the article and approved the submitted version.

## Funding

This study was supported by grants from the National Natural Sciences Foundation of China (Nos. 81800132 and 81973995) and Program for HUST Academic Frontier Youth Team (No. 2018QYTD14).

## Conflict of Interest

The authors declare that the research was conducted in the absence of any commercial or financial relationships that could be construed as a potential conflict of interest.

## Publisher's Note

All claims expressed in this article are solely those of the authors and do not necessarily represent those of their affiliated organizations, or those of the publisher, the editors and the reviewers. Any product that may be evaluated in this article, or claim that may be made by its manufacturer, is not guaranteed or endorsed by the publisher.
